# Mating dynamics and multiple paternity in a long‐lived vertebrate

**DOI:** 10.1002/ece3.5438

**Published:** 2019-09-05

**Authors:** Joshua Zajdel, Stacey L. Lance, Thomas R. Rainwater, Phillip M. Wilkinson, Matthew D. Hale, Benjamin B. Parrott

**Affiliations:** ^1^ Odum School of Ecology University of Georgia Athens GA USA; ^2^ Savannah River Ecology Laboratory Aiken SC USA; ^3^ Tom Yawkey Wildlife Center & Belle W. Baruch Institute of Coastal Ecology and Forest Science Clemson University Georgetown SC USA; ^4^ Tom Yawkey Wildlife Center Georgetown SC USA

**Keywords:** American alligator, mate selection, multiple paternity, reproductive success

## Abstract

Multiple paternity is relatively common across diverse taxa; however, the drivers and implications related to paternal and maternal fitness are not well understood. Several hypotheses have been offered to explain the occurrence and frequency of multiple paternity. One set of hypotheses seeks to explain multiple paternity through direct and indirect benefits including increased genetic diversity or enhanced offspring fitness, whereas another set of hypotheses explains multiple paternity as a by‐product of sexual conflict and population‐specific parameters such as density. Here, we investigate mating system dynamics in a historically studied population of the American alligator (*Alligator mississippiensis*) in coastal South Carolina. We examine parentage in 151 nests across 6 years and find that 43% of nests were sired by multiple males and that male reproductive success is strongly influenced by male size. Whereas clutch size and hatchling size did not differ between singly sired and multiply sired nests, fertility rates were observed to be lower in multiply sired clutches. Our findings suggest that multiple paternity may exert cost in regard to female fitness, and raise the possibility that sexual conflict might influence the frequency of multiple paternity in wild alligator populations.

## Graphical abstract

We examine parentage in 151 nests across 6 years and find that 43% of nests were sired by multiple males and that male reproductive success is strongly influenced by male size. Whereas clutch size and hatchling size did not differ between singly sired and multiple sired nests, fertility rates were observed to be lower in multiply sired clutches.

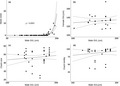

## 1 INTRODUCTION

One of the most surprising discoveries resulting from modern genetic analysis of mating systems is that multiple paternity, wherein more than one male sires a clutch or litter, is relatively common across vertebrates (Birkhead & Møller, 1998; Coleman & Jones, 2011; Griffith, Owens, & Thuman, 2002; Uller & Olsson, 2008). However, evolutionary explanations for the occurrence of multiple paternity and whether it is adaptive for females are not always evident (Birkhead & Møller, 1998; Griffith et al., 2002; Jennions & Petrie, 2000; Uller & Olsson, 2008). Hypotheses explaining variation in the frequency of multiple paternity typically include direct or indirect benefits to the female, wherein direct benefits encompass male contribution to parental care, improved genetic quality of offspring, and increased fertilization success (see reviews Birkhead & Møller, 1998; Griffith et al., 2002; Jennions & Petrie, 2000; Uller & Olsson, 2008). Indirect benefits can stem from promoting sperm competition, cryptic female choice, or genetic bet‐hedging to create a genetically diverse clutch (Eberhard, 1996; Jennions & Petrie, 2000; Keller & Reeve, 1995; Yasui, 1998). All of these explanations suggest that multiple mating by females is adaptive. Contrary to this idea, it has also been suggested that multiple paternity might result from sexual conflict and be nonadaptive for females (Andersson, 1994; Arnqvist & Kirkpatrick, 2005; Lee & Hays, 2004). Under this scenario, the number of matings by females may increase with mate encounter rate and be limited by the cost of mating to females (Andersson, 1994).

In nonavian reptiles, there is broad support for multiple paternity resulting from female mating frequency being driven by mate encounter rates (Fitze, Galliard, Federici, Richard, & Clobert, 2005; Garner et al., 2002; Jensen, Abreu‐Grobois, Frydenberg, & Loeschcke, 2006; Laloi, Richard, Lecomte, Massot, & Clobert, 2004; Lee & Hays, 2004; Olsson & Shine, 1997). Although exceptions exist, nonavian reptiles typically do not provide paternal care, and therefore, offspring would not benefit from increased care from multiple males (Gans, 1996). Furthermore, some studies have failed to find evidence for direct or indirect benefits from multiply paternity (Byrne & Robert, 2000; Fitze et al., 2005; Garner et al., 2002; Jensen et al., 2006; Laloi et al., 2004; Lee & Hays, 2004; Olsson & Shine, 1997). For example, studies on multiple paternity among Australian myobatrachid frogs (*Crinia georgiana*) found no significant advantage to offspring from multiply sired clutches (Byrne & Robert, 2000). However, other studies report correlations between population density and the frequency of multiple paternity (Fitze et al., 2005; Jensen, et al., 2006; Laloi et al., 2004; Lee & Hays, 2004). In olive ridley sea turtles (*Lepidochelys olivacea*), the frequency of multiple paternity varies across nesting sites, with nesting sites having higher densities of turtles also characterized by higher frequencies of multiple paternity (Jensen et al., 2006). However, given the taxonomic and behavioral diversity in nonavian reptiles, there is still debate as to the drivers of multiple paternity (Byrne & Robert, 2000; Fitze et al., 2005; Jensen et al., 2006; Laloi et al., 2004; Lance et al., 2009; Lee & Hays, 2004; Olsson & Shine, 1997).

Crocodilians, which have widely varying population densities and degrees of male territoriality, provide an excellent system to explore the evolutionary and ecological drivers that underlie the observed variation in the frequency of multiple paternity (Amavet, Rosso, Markariani, & Piña, 2008; Budd, Spotila, & Mauger, 2015; Davis, Glenn, Elsey, Dessauer, & Sawyer, 2001; Lance et al., 2009; Lewis, FitzSimmons, Jamerlan, Buchan, & Grigg, 2013; Mcvay et al., 2008; Muniz et al., 2011; Ojeda, Amavet, Rueda, Siroski, & Larriera, 2017; Oliveira, Marioni, Farias, & Hrbek, 2014; Lafferriere et al., 2016; Wu & Hu, 2010). The frequency of multiple paternity observed across crocodilian taxa ranges from 32% in the Chinese alligator (*Alligator sinensis*) to 100% in black caiman (*Melanosuchus niger*) (Muniz et al., 2011; Wu & Hu, 2010). Among crocodilians, it is not clear if the frequency of multiple paternity is driven by population density and/or mate encounter rate (Amavet et al., 2008; Budd et al., 2015; Davis et al., 2001; Lance et al., 2009; Lewis et al., 2013; McVay et al., 2008; Muniz et al., 2011; Oliveira et al., 2014; Lafferriere et al., 2016; Wu & Hu, 2010) though both have been suggested (Budd et al., 2015; Lafferriere et al., 2016).

The most thoroughly studied crocodilian species in terms of multiple paternity and mating behavior is the American alligator (*Alligator* *mississippiensis*) (Davis et al., 2001; Garrick & Lang, 1977; Joanen & McNease, 1971; Lance et al., 2009). However, because observing mating activity in the wild is difficult, most research into mate selection dynamics has focused on captive populations (Garrick & Lang, 1977; Joanen & McNease, 1971). Studies on these animals describe a complex courtship process with larger male alligators holding territories more successfully than smaller males (Garrick & Lang, 1977; Joanen & McNease, 1971). However, whether territorial gains translate into reproductive success remains unknown.

Previous studies investigating mating dynamics of wild alligators using genetic techniques have exclusively examined the population at the Rockefeller National Wildlife Refuge (RNWR) in Louisiana (Davis et al., 2001; Lance et al., 2009). These studies found that an average of 46% of observed nests have multiple sires (Davis et al., 2001; Lance et al., 2009). The study by Lance et al. (2009) was also the first to demonstrate mate fidelity across years in any crocodilian species. However, in both studies males were identified solely by offspring genotypes, and thus, the phenotypic attributes of males that might lead to higher reproductive success could not be inferred. Wild alligators examined in this investigation are part of a long‐studied population (Wilkinson, Rainwater, Woodward, Leone, & Carter, 2016) for which data on size, sex, and age of many individuals are available. Here, we examine mating dynamics in the American alligator with respect to the frequency of multiple paternity, the role of male characteristics in male reproductive output, and potential fitness benefits to females with multiply sired clutches. By examining these questions within the context of the American alligator mating system, we seek to better understand whether multiple paternity is driven by evolutionary fitness advantages across sexes or is the product of population‐specific parameters.

## 2 METHODS

### 2.1 Site description

This study was conducted on the South Island and Cat Island portions (6,033 ha) of the Thomas A. Yawkey Wildlife Center (YWC), a wildlife management area operated by the South Carolina (SC) Department of Natural Resources. The YWC alligator population is relatively closed, in that it is bordered by saltwater on all sides: the Atlantic Ocean to the east, Winyah Bay to the north, the Intracoastal Waterway to the west, and North Santee Bay to the south. This alligator population is well characterized due to long‐term (1970s to present) mark–recapture efforts resulting in a large database of alligator tissue, nesting, and morphometric data (Hale et al., 2017; McCoy, Parrott, Rainwater, Wilkinson, & Guillette, 2015; Parrott et al., 2014; Wilkinson et al., 2016).

### 2.2 Egg and hatchling collection

Alligator eggs were collected at YWC from 2011 to 2017. Weekly helicopter surveys were used to locate nests from the air during the alligator nesting season in SC (early June–early July; Wilkinson, 1984). Nests were visited daily on foot until oviposition was confirmed**.** Fertility rates were determined by observing banding patterns (fertile eggs exhibit an opaque patch or band on the eggshell; Ferguson, 1982). Clutch fertility rates were quantified as the proportion of eggs within the nest that were viable according to their banding pattern. Eggs were collected within 48 hr of oviposition and transported to the Hollings Marine Laboratory (2011–2016) in Charleston, SC, or the University of Georgia Savannah River Ecology Laboratory (2017) in Aiken County, SC, where they were either necropsied as embryos or reared to hatching. In some cases, entire clutches of eggs were taken, while at other nests only a subset (1–8 eggs) was collected (Table 1).

**Table 1:** The number of clutches and hatchlings sampled during each year of the study and the results regarding multiple paternity

Year	Total clutches sampled	Full clutches	Hatchlings collected	Multiply sired clutches (%)
2011	10	0	66	—
2012	11	8	267	2 (25%)
2013	20	9	305	4 (44%)
2014	19	4	110	3 (75%)
2015	19	0	135	—
2016	44	0	319	—
2017	28	10	455	3 (30%)
Total	151	31	1657	12

In 2012, 2013, and 2017, twenty‐seven full clutches were collected, maintained in damp sphagnum moss, and reared until hatching. For all years in which eggs were allowed to hatch, eggs were checked twice daily for the initiation of hatching, and once hatchlings had pipped, they were removed from sphagnum and transferred to individual glass jars. Neonates were weighed, and snout–vent length (SVL), total length, cloacal tail girth, and both head and snout length and width were measured. Scutes and/or chorioallantoic membrane were also collected shortly after hatching. All tissue samples collected from hatchling alligators were immediately stored at −20°C upon collection. A total of 1,657 hatchlings were sampled from 151 nests. For 31 nests, we collected the entire clutch of eggs. For the remaining 120 nests, a subset of the eggs were collected (1–8 eggs).

### 2.3 Adult alligator capture and sampling

Adult alligators were captured using multiple methods including baited‐trip snare traps, walk‐through traps, snare poles, and snatch hooks (Cherkiss, Fling, Mazzotti, & Rice, 2004; Murphy, Wilkinson, Coker, & Hudson, 1983; Wilkinson, 1994; Wilkinson et al., 2016). Over the course of the study, we sampled 204 adult alligators, 120 females and 84 males. Of the 120 females sampled, 76 were captured on or near a nest. Alligator SVL ranged from 63.5 to 176.0 cm (females) and from 73.66 to 194.3 cm (males). The preponderance of females in our data set was the result of a research focus on nesting ecology from 2009 to 2017 in which female alligators were captured at their nests (Wilkinson et al., 2016). Following capture, total length, SVL, and tail girth were measured for each animal and scute and blood samples collected. All samples collected in the field were stored on ice until transport to the laboratory where they were stored at −20°C until DNA extraction.

### 2.4 DNA extraction

Alligator DNA was isolated from a variety of sample types including adult blood and scutes, hatchling chorioallantoic membranes, scutes, and embryos preserved in RNAlater. DNA isolation was performed using the DNeasy blood and tissue kit (Qiagen) following the manufacturer's protocols with the following exceptions. EconoSpin columns (Epoch Life Sciences, Inc.) were used during DNA filtration, and DNA was eluted with 100 µl of the provided AE buffer. DNA concentrations were determined using a NanoDrop Spectrophotometer ND‐1000 (Thermo Scientific) and standardized to 20 ng/µl.

### 2.5 Microsatellite development

We initially screened a subset of samples using the same microsatellite loci used by Lance et al. (2009). However, the YWC samples exhibited insufficient genetic variation for conducting parentage analyses, so new microsatellite loci were developed (see Appendix S1 for a full description).

### 2.6 Maternal genotype comparison and genotyping error rate

Hatchling alligator genotypes were initially screened using the program Gerud 2.0 to test that each clutch could be explained by a single maternal genotype (Jones, 2005). The genotypes of clutches that could not be explained by a single maternal genotype were examined for unexpected alleles. If an unexpected allele occurred at one locus, the allele was considered to be a mutation and the allele calls for that hatchling at the locus were excluded from future analysis. If an individual contained two or more alleles that prevented the clutch from having a single maternal genotype, the individual was removed from further analysis. Almi 40 consistently produced unreliable alleles and was removed from future analysis.

Following the initial screening process, hatchling genotypes were compared to the genotype of the female caught at the nest to confirm maternity. If the genotype of a female captured at a nest was not consistent with maternity, then the female DNA and hatchling DNA were re‐extracted and the female's microsatellite loci were amplified in triplicate and hatchling microsatellite loci were amplified in duplicate. Allele calls from the same individual but different amplifications were compared in order to estimate the genotyping error rate. A total of 457 individuals (28% of the total number of individuals in the study) were reanalyzed to determine the genotyping error rate. Almi 19, Almi 32, Almi 39, and Almi 46 all had genotyping error rates above 10% and were therefore excluded from further analysis. The remaining loci had an average genotyping error rate of 5% with a standard deviation of 2% (Table 2). Table 2 shows the number of alleles per locus (*k*), observed and expected heterozygosity (*H*_o_ and *H*_e_), mean polymorphic information content (PIC), the nonexclusion probability for the first parent (NE‐1P), the nonexclusion probability for the second parent (NE‐2P), and the nonexclusion probability for the parent pair (NE‐PP) for the remaining loci that were used in parentage assignment and multiple paternity detection.

**Table 2:** Details on the loci used for parentage analysis and multiple paternity detection

Loci	*k*	*H*_o_	*H*_e_	PIC	NE‐1P	NE‐2P	NE‐PP	Error rate
Almi 8	12	0.81	0.814	0.791	0.530	0.355	0.169	0.04
Almi 26	11	0.797	0.815	0.789	0.539	0.364	0.183	0.02
Almi 30	20	0.839	0.841	0.822	0.476	0.31	0.134	0.07
Almi 34	15	0.813	0.851	0.833	0.458	0.296	0.125	0.08
Almi 47	9	0.667	0.67	0.627	0.732	0.557	0.362	0.06
Total	—	—	—	—	0.046	0.0066	0.00188	0.05

Note

*H*_o_ is the observed heterozygosity, *H*_e_ is the expected heterozygosity, PIC is the mean polymorphic information content, NE‐1P is the nonexclusion probability for the first parent, NE‐2P is the nonexclusion probability for the second parent, and NE‐PP is the nonexclusion probability for the parent pair.

### 2.7 Parentage assignment

We used Cervus 3.0.7 to assign parentage (Kalinowski, Taper, & Marshall, 2007). We ran an initial simulation with 10,000 offspring, the estimated 5% genotyping error rate, and with 90% of all loci having allele calls in Cervus to calculate the confidence of each parental assignment. Confidence intervals were set to 80% (relaxed) and 95% (strict). When assigning maternity, if Cervus assigned a single female to the majority of hatchlings from a single nest with a high logarithm of the odds score (LOD), then the genotype of the proposed female was compared to the hatchling genotypes. If the proposed female genotype was consistent with maternity for 90% of the hatchling allele calls, then we assigned the female as the mother of the clutch. Paternity assignments were made based off the LOD scores. If Cervus proposed the same male to have sired multiple individuals within a clutch and those matches fell within the strict 95% confidence interval range, then the male genotype was compared to the clutch genotypes to determine which hatchlings within the clutch were fathered by the proposed male. Less strict criteria were used for paternity assignments in order to allow for the possibility of multiple paternity and multiple males being assigned to a single nest.

### 2.8 Multiple paternity detection

Multiple paternity was detected by two separate methods. In clutches for which maternity was known, allelic counting was used to determine if multiple paternity occurred. For nests without a known mother, the program Colony was used to determine intraclutch relatedness as well as the likely number of sires (Jones & Wang, 2010). Colony uses a maximum‐likelihood full‐pedigree analysis to assign individuals into either full‐sibling or half‐sibling categories (Jones & Wang, 2010). If a clutch contains individuals who are half‐siblings, then multiple paternity is determined to have occurred (Jones & Wang, 2010; Lafferriere et al., 2016). Colony runs were conducted under the “high precision” likelihood while incorporating the estimated genotyping error rate of 5%.

Our power to detect multiple paternity was tested with Gerudsim 2.0 (Jones, 2005). Gerudsim 2.0 uses provided allele frequencies, clutch size, number of males contributing to a clutch, the number of offspring sired by each male, and whether or not the maternal genotype is known to simulate potential clutch genotypes, maternal genotypes, and paternal genotypes. These simulated genotypes are then passed to Gerud 2.0 to test if Gerud 2.0 is able to accurately recreate the correct paternal and maternal genotypes (Jones, 2005). We simulated 39 egg clutches sired by three males with one male contributing to 24 eggs, another male contributing to 10 eggs, and the final male contributing to 5 eggs. With a known mother, 11 eggs needed to be sampled in order to accurately recreate the paternal genotypes 75% of the time. Without a known mother and 11 eggs sampled, we were able to accurately recreate the paternal genotypes 70% of the time. As a result, our estimates of multiple paternity are likely to be underestimates.

### 2.9 Statistical analysis

All statistical analyses were performed using R statistical software version 3.4.0 (R Development Core, 2017). Generalized linear mixed modeling (GLMM) was used to assess models where either nest counts, clutch size, or the presence of multiple paternity was a response variable. Models in which clutch size was the response variable were run with a Poisson's error distribution. Models in which the presence of multiple paternity was the response variable were run with a binomial error distribution. Zero‐inflated GLMMs were used to assess the influence of male morphometric characteristics on the number of nests sired. Zero‐inflated models were performed using the function “zerinfl” from the package MuMIN (Barton & Barton, 2015). The effects of male morphometrics on the number of nests sired were compared using Akaike information criterion with a correction for small samples size (AICc) as well as by using Akaike weights (Burnham & Anderson, 2002). AICc values were calculated using the “AICc” function within the package MuMIN (Barton & Barton, 2015). Linear models were used to assess the influence of male size on clutch fertility and the influence of multiple paternity on clutch fertility. The influence of multiple paternity on hatchling mass, the influence of multiple paternity on hatchling SVL, and the influence of multiple paternity on hatchling body condition were examined independently using linear mixed modeling where clutch identity was included as a random effect. These models were run using the function lmer from the package “lme4” (Bates et al., 2007). *p*‐values were extracted from these models using the function summary from the “lmerTest” R packages (Kuznetsova et al., 2017). Within R, the function “rcorr” within the package Hmisc was used to perform a Pearson's correlation test on maternal size and paternal size (Harrell & Dupont, 2008). The function “moran.I” within the R package lctools to perform a global Moran's I test was used to determine the degree of spatial autocorrelation between multiply sired and singly sired nests (Kalogirou, 2016). For nests with multiple paternity, a Wilcoxon ranked sum test was used to compare the contributions from the primary males and secondary males at nests sired by two or three males. All variables were considered significant at *p*‐values of less than 0.05.

## 3 RESULTS

### 3.1 Parentage and clutch characteristics

Of the 151 nests examined, we assigned a mother to 78 and at least one father to 38. For 28 nests, we assigned both maternity and paternity. The majority of maternity assignments matched the female that was caught at the nest (81%). However, at 15 nests, the female captured at the nest was determined not to be the maternal female. Three pairs of alligators were found to have mated with each other across multiple years (Table 2). No cases of multiple paternity were detected within nests that had been sired by the same pair across years.

**Figure 1:**
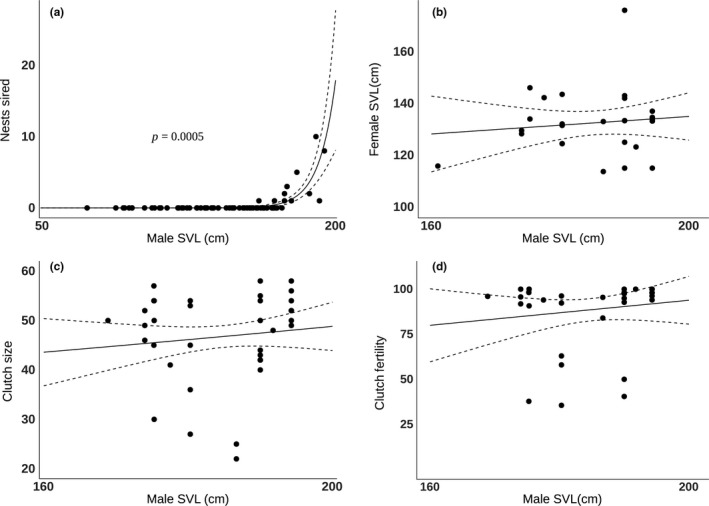
Relationships between male SVL and (a) the number of nests sired, (b) size of female mate, (c) clutch size, and (d) clutch fertility

Only 12 males contributed to the 38 nests for which paternity assignments were made, and two males sired 47% of these nests (Figure 1). In order to identify factors that may be causing these males to sire such a large percentage of the nests, we examined the relationship between male size and number of nests sired. When modeled separately, SVL, total length, and tail girth were all found to be significantly related to the number of nests sired (SVL: *z*‐value = −2.251, *p* = 0.02, total length; *z*‐value = −2.730, *p* = 0.01; tail girth: *z*‐value = 2.719, *p* = 0.01). Interestingly, neither the ratio of tail girth to SVL nor the ratio of tail girth to total length was a significant predictor of the number of nests sired, indicating that length, but not proxies for body condition, correlated with male mating success. Upon comparing AICc values among models, SVL was a factor in the top two models (Table 3). The top model was SVL plus the additive effect of tail girth and SVL alone (Table 3). Interestingly, tail girth was no longer significant as an additive effect within the top performing model (Table 3).

**Table 3:** An AICc table of the AICc scores, Delta AICc and model weight or each model used to examine the effect of male morphometrics on the number of nests each male sired

Formula	AICc	ΔAICc	Weight
Nest Sired ~ SVL + Tail Girth	72.8818	0	0.56
Nest Sired ~ SVL	73.38822	0.50642	0.44
Nest Sired ~ Total Length + Tail Girth	87.31301	14.43121	0
Nest Sired ~ Tail Girth	89.35854	16.47674	0
Nest Sired ~ Total Length	94.09322	21.21142	0
Nest Sired ~ Ratio of SVL to Tail Girth	125.482	52.6002	0
Nest Sired ~ Ratio of Total Length to Tail Girth	125.8006	52.9188	0

Male size was not related to clutch fertility (*t*‐value = −0.582, *p* = 0.56; Figure 1) nor clutch size (*z*‐value = 0.935, *p* = 0.35; Figure 1). We next tested if larger males mated with larger females, but detected no significant correlation between paternal and maternal size (Pearson's correlation coefficient = 0.12, *p* = 0.54; Figure 1). Multiple paternity was confirmed for only three nests for which a known male was identified as the sire of the nest; therefore, the relationship between male size and multiple paternity could not be examined. Together, these data suggest that length is a key determinant of male reproductive success.

### 3.2 Multiple paternity

**Figure 2:**
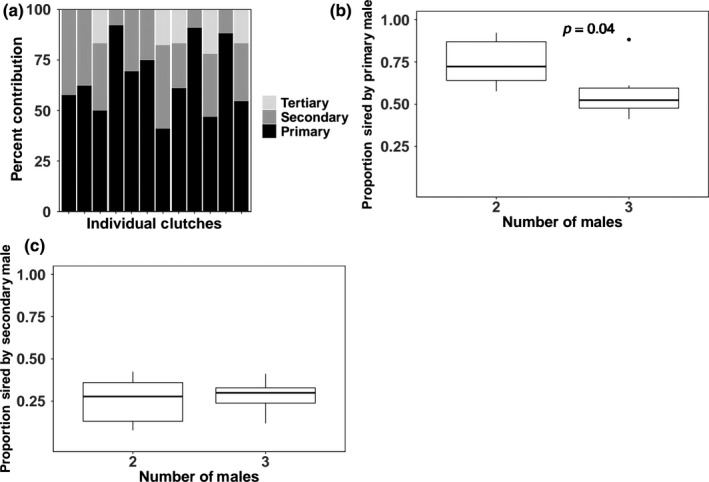
Examination of male contributions to nests with (a) the distributions of contributions across primary, secondary, and tertiary males, (b) the distribution of contributions across primary males, and (c) the distribution of contributions across secondary males

Based on our simulations, we determined that the probability of accurately detecting the number of sires when we collected eight or fewer eggs was less than 70%. Therefore, we excluded 116 nests with eight or fewer eggs from our analyses of multiple paternity. This removed all nests collected in 2011, 2015, and 2016. We detected multiple paternity at 12 (35%) of the remaining 35 nests, and rates of multiple paternity varied across years with an average of 43.5% per year (Table 1). Within multiply sired nests, we detected up to three males contributing to a clutch. For 80% of multiply sired nests, there was a primary male that was responsible for ≥50% of the hatchlings in the clutch (Figure 2a). We next asked if paternal contribution from a tertiary male detracts from the proportion of eggs sired by either the primary or secondary male. Interestingly, the primary male sired an average of 74.5% of the clutch when there were two sires, but only 57% in the presence of a tertiary sire (*w* = 31, *p* = 0.04; Figure 2b). However, the presence of a tertiary male did not affect the proportion of sired offspring from the secondary male (*w* = 16, *p* = 0.82; Figure 2c).

**Figure 3:**
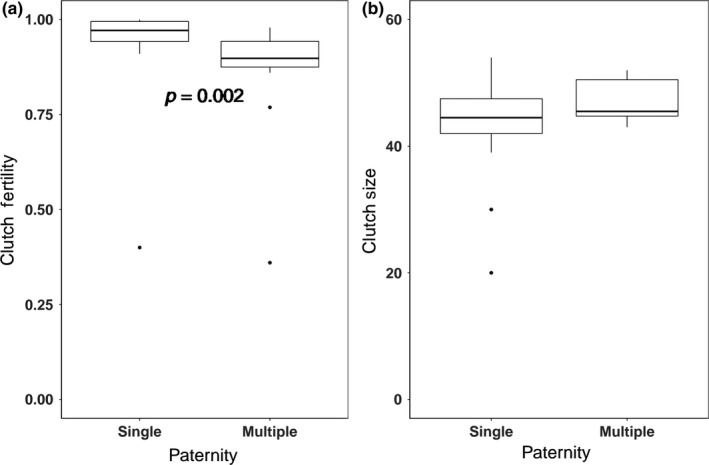
Relationships between fitness‐related traits and multiple paternity including (a) clutch fertility and (b) clutch size across singly sired and multiply sired nests

We next examined how multiple paternity might influence clutch characteristics. The occurrence of multiple paternity was not correlated with clutch size (*w* = 70.5, *p* = 0.12; Figure 3). However, clutch fertilization rates (percentage of fertilized eggs) were significantly greater in nests with only one sire (94%) when compared to those that were multiply sired (86%, *w* = 179, *p* < 0.01; Figure 3). Further, we reasoned that fertility rates and the frequency of multiple paternity might be indirectly linked by maternal traits. However, female size was not correlated with the frequency of multiple paternity or fertilization rates, suggesting that multiple paternity might confer a direct fitness cost to maternal females in terms of reduced fertilization rates (female size and fertility: *t* = 0.257, *p* = 0.80; female size and multiple paternity: *t* = 0.528, *p* = 0.61).

**Figure 4:**
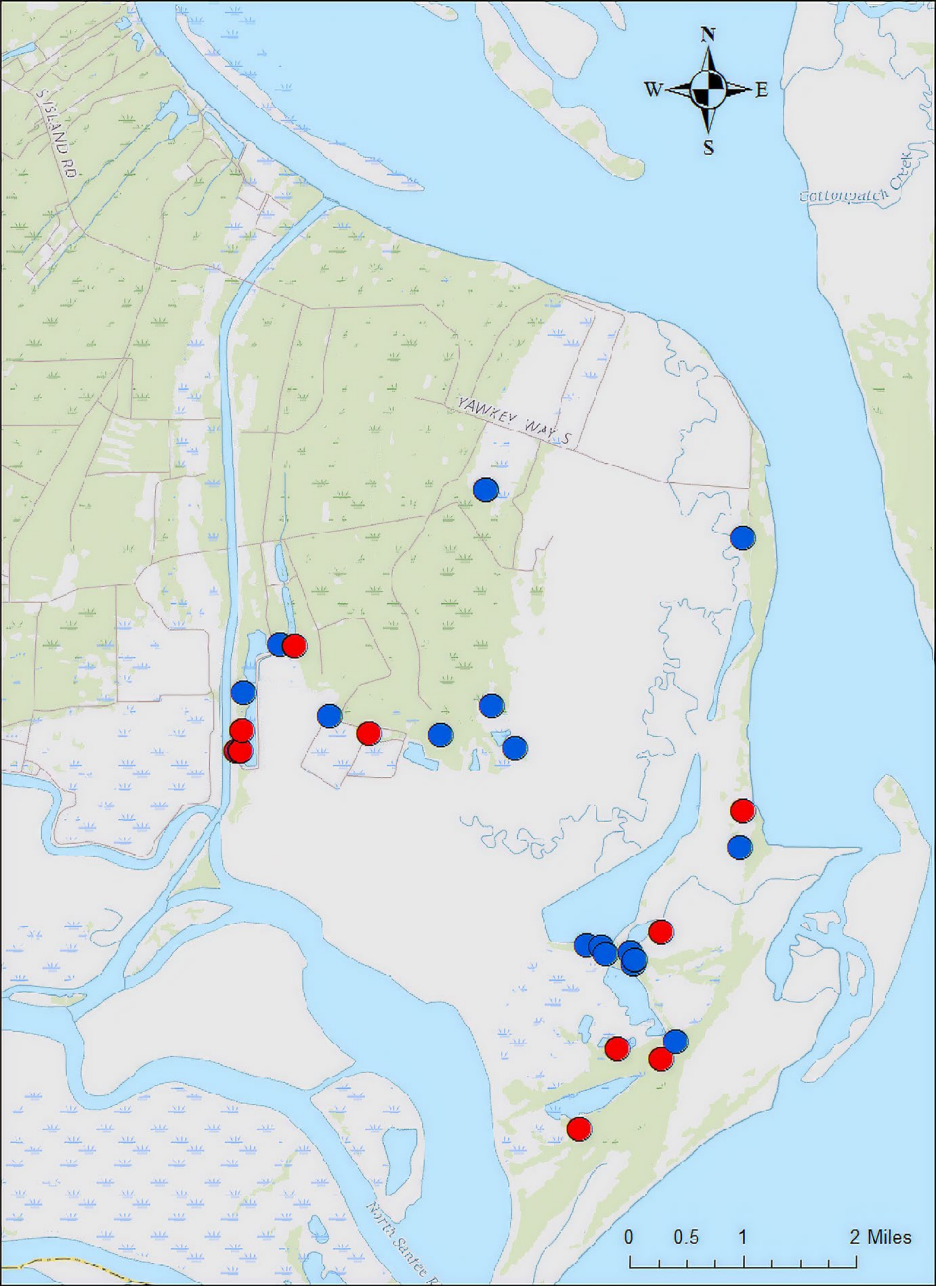
Map of YWC with points indicating nests for which the entire clutch was sampled (*N* = 31). Blue points represent nests that were singly sired, and red points represent nests that were multiply sired

We next asked if the frequency of multiple paternity might be influenced by landscape characteristics and examined the spatial orientation of singly sired and multiply sired nests. We found that multiply sired nests were not clustered with other multiply sired nests, nor were singly sired nests found to cluster with singly sired nests (Moran's I = −0.069, *z*‐randomization = −0.36, *p*‐randomization = 0.71; Figure 4). However, more detailed analyses are required to determine if landscape characteristics, such as habitat type and quality, associated with nest site might influence the mating dynamics underlying the frequency of multiple paternity.

### 3.3 Implications of multiple paternity on offspring phenotype

**Figure 5:**
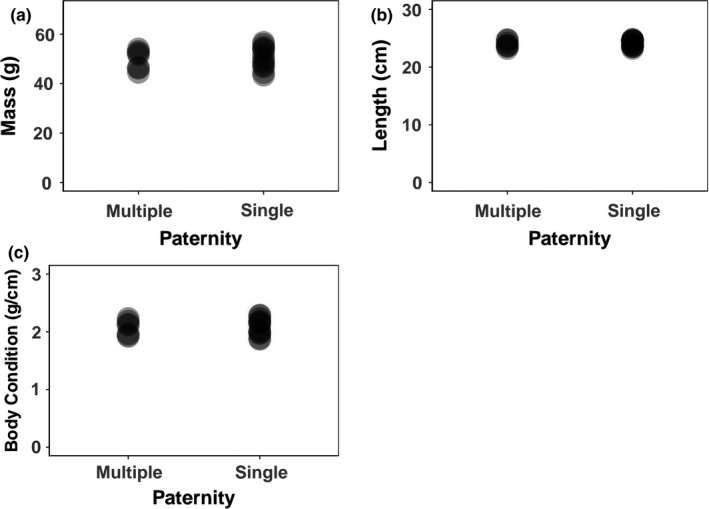
Relationships between hatchling phenotypes and patterns of paternity with (a) hatchling mass, (b) hatchling length, and (c) body condition across singly sired and multiply sired nests

In an effort to further explore the potential benefits and fitness costs associated with multiple paternity, we examined whether multiple paternity influences hatchling phenotypes. We compared the body mass and SVL of hatchlings from 21 complete clutches collected in 2012, 2013, and 2017. No significant differences were found between the hatchling sizes from multiply sired and singly sired nests in terms of mass, length, or body condition (mass: *t*‐value = 1.11, *p* = 0.28; length: *t*‐value = 1.21, *p* = 0.24; body condition: *t*‐value = 1.01, *p* = 0.33; Figure 5).

## 4 DISCUSSION

It is well documented that large male alligators are better able to establish and maintain territories when compared to smaller males (Garrick & Lang, 1977; Joanen & McNease, 1971). However, how these territorial advantages influence a male alligator's reproductive output is not known. The current study presents strong evidence that larger males sire more nests. Interestingly, these larger males do not sire larger nor more fertile clutches, suggesting that territorial advantages of larger males translate into more mating opportunities but perhaps not higher quality mates. In captive studies, female alligators were found to preferentially mate with larger males (Joanen & McNease, 1971). This appears to extend to wild populations as we saw no size‐assortative mating but did find that only males >2.86 m in total length sired offspring. In Louisiana, male alligators as small as 1.83 m in total length produce sperm during the mating season (Joanen & McNease, 1980), and it is possible that while these males are physiologically cable of mating, they are excluded from entering into the breeding population by larger males or by female selection (Garrick & Lang, 1977; Hamlin et al., 2011; Joanen & McNease, 1971). Adult males with an SVL of 135 cm or less display seasonal increases in testosterone (T), similar to larger males, until late March, after which T concentrations in smaller males decrease, whereas T concentrations in larger males continue to increase into April (breeding season) and remain much higher through June (Hamlin et al., 2011). This physiological observation is consistent with smaller males being excluded from the breeding population and is perhaps mediated through social interactions with larger, more dominant males.

Our study is the first to describe multiple paternity in the American alligator outside of Louisiana (RNWR). Multiple paternity occurred in 25%–75% of nests examined from 2012 to 2017 with an average of 43% of examined nests in a year having multiple paternity. These estimates align closely with the frequency of multiple paternity observed at RNWR (46.6%). Despite this similarity in occurrence of multiple paternity, these sites are characterized by substantial ecological differences. Whereas RNWR is dominated by open marsh, YWC is a series of coastal islands fragmented into diverse habitat types (Coates et al., 2018; Joanen, 1969; Obernuefemann, Collazo, & Lyons, 2013; Wilkinson et al., 2016), suggesting that habitat characteristics may not be an important determinant of multiple paternity frequency across American alligator populations.

Uller and Olsson (2008) suggest that within the nonavian reptiles, the occurrence of multiple paternity may reflect the number of males encountered by a female during her reproductive cycle. This density‐driven pattern may be true in other nonavian reptiles. Studies on the common garter snake (*Thamnophis sirtalis*) found higher rates of multiple paternity in a population associated with larger communal hibernation and mass‐mating behavior (Garner et al., 2002). This pattern may be true for alligators as well. Female alligators increase home range size and movement during the spring mating season and have the potential to contact multiple males (Garrick & Lang, 1977; Goodwin & Marion, 1979; Rootes & Chabreck, 1993). However, estimates for population size and density are not available for either YWC or RNWR, and without these estimates or male–female encounter rates, this hypothesis cannot be directly examined. The occurrence of multiple paternity may also be mediated through sex ratios. One study on the common lizard (*Zootoca vivipara*) found that the number of mating partners a female had increased in male‐biased enclosures (Fitze et al., 2005). A study on the sex ratios of American alligator populations found that wild alligator populations are generally male‐biased (Lance, Elsey, & Lang, 2000). However, this study also noted year‐to‐year variation, with one year being female‐biased (Lance et al., 2000). If the occurrence of multiple paternity in the American alligator is driven by the population's sex ratios, then the year‐to‐year variation in sex ratios within an alligator population may correspond with the year‐to‐year variation in multiple paternity.

This study, as well as those by Lance et al. (2009) and Davis et al. (2001), found no more than three male alligators contributing to a single clutch. All three studies also found that the contribution of the primary male, but not the secondary male, decreases in the presence of a tertiary male. This pattern of paternal contribution might reflect the number of copulation events during ovulation. Because each successive male's contribution to a clutch comes at the expense of the primary male, it is tempting to speculate that paternal contribution of a secondary and tertiary male result from a single mating event. Under this scenario, the primary male maintains a territory to increase the frequency of copulation events and experiences strong evolutionary pressure to prevent other males from contributing to a clutch (Emlen & Oring, 1977). This may lend further support to the idea that the reproductive advantage of larger size in male alligators is the increased ability to hold a territory and exclude other males' access to females within that territory. An alternative possibility is that the loss of paternal contribution from the primary male reflects the primary male's inability to completely fertilize the clutch. However, multiple paternity would be expected to increase fertilization rates under this scenario, which is the opposite of what we observed. American alligators do have the ability to store sperm within a breeding season, and thus, the potential for sperm competition exists (Gist, Bagwill, Lance, Sever, & Elsey, 2008). To date, no studies have examined sperm competition in alligators; therefore, the role of male sperm quality in multiple paternity in these animals is unknown.

We found that hatchling alligators from multiply sired clutches were not significantly different in terms of mass, length, or body condition when compared to hatchlings from singly sired nests. These findings do not support a role for multiple paternity in increasing fitness through benefits to offspring. However, the implications of hatchling size in alligators in terms of long‐term fitness or survival are currently unclear, and other studies have documented increases in fitness‐related traits in the offspring of other species resulting from multiply sired clutches (see reviews Griffith et al., 2002; Jennions & Petrie, 2000). Costs or a lack of benefit to hatchling fitness as a result of multiple paternity is a predicted outcome if multiple paternity is primarily a product of male harassment (Fitze et al., 2005; see review Uller & Olsson, 2008). Studies on the common lizard (*Z. vivipara*) have shown that females in male‐biased enclosures have decreased reproductive output despite mating with more males as detected through mating scars (Fitze et al., 2005). Male harassment could explain multiple paternity in American alligators given that we observed decreases in clutch fertility indicating an overall cost to females of mating multiply. Contrary to this idea are other observational studies indicating that female alligators are able to reject male advances and will even kill potential male suitors (Garrick & Lang, 1977; Joanen & McNease, 1971), though in these studies the rejected or killed males were smaller than the males we detected within the breeding population at YWC (Garrick & Lang, 1977; Joanen & McNease, 1971). It is possible that once a male reaches a certain size, females are no longer able to avoid mating. The role of male harassment within American alligator mating dynamics remains unclear and requires further study.

Our study was able to document three cases in which the same parent pair sired nests across years. These results are similar to the findings of Lance et al. (2009) with the exception that our study found no cases of mate fidelity and multiple paternity within the same clutches. Mate fidelity is often explained with three hypotheses: Males assist in parental care in order to increase their own reproductive success, males defend females from rival males to ensure paternity, or females adopt monogamy in order to gain some advantage from the male (Bull, 2000). Male parental care has not been documented in the American alligator, and while males will defend a territory, females will interact with multiple males during a breeding season (Garrick & Lang, 1977; Joanen & McNease, 1971).

At YWC, larger males are better able to maintain territorial advantages and we show they are also able to sire more nests (Garrick & Lang, 1977; Joanen & McNease, 1971). Together, our work and the work of previous researchers suggest that the advantage of size and territory translates into more mating opportunities for male alligators. Further, multiple paternity led to a decrease in clutch fertility, but had no impact on those hatchling phenotypes observed. These results are inconsistent with hypotheses in which multiple paternity results in benefits to females or offspring (Arnqvist & Kirkpatrick, 2005; Birkhead & Møller, 1998; Bull, 2000; Byrne & Robert, 2000; Eberhard, 1996; Laloi et al., 2004; Lee & Hays, 2004; Olsson & Shine, 1997). However, our findings are consistent with a system in which multiple paternity is the product of sexual conflict (Fitze et al., 2005; Jensen et al., 2006). Thus, this study advances our understanding into the evolutionary and ecological drivers of mating system diversity, particularly in the context of long‐lived vertebrates.

## CONFLICT OF INTEREST

None declared.

## AUTHORS' CONTRIBUTION

JZ performed experiments, analyzed and interpreted results, and wrote the initial manuscript; SL contributed to the development of the microsatellite markers, genetic analyses, and interpretation of results and helped write the manuscript; TR contributed to the study design, supervised field collections, and contributed to the writing of the manuscript; PW contributed to study design, led fieldwork, maintained the database of all alligator morphometric data and nest data, and reviewed and edited the manuscript prior to submission; MH contributed to experiments and reviewed and edited the manuscript prior to submission; and BP contributed to the study design, project supervision, field collections, analyses of findings, and writing of the manuscript.

## ETHICAL APPROVAL

All data were collected under UGA IACUC approval: A2017 05‐005‐Y2‐A0. Field data were collected under SCDNR Scientific Collection Permit SC‐04‐2017.

## Data Availability

All data used in the manuscript can be accessed at the Dryad Digital Data Repository using https://doi.org/10.5061/dryad.34gf741.

## APPENDIX A

### Microsatellite Development

We extracted DNA from one individual and prepared an Illumina paired‐end shotgun library by shearing 1 µg of DNA using a Covaris S220 sonicator and following the standard protocol of the Illumina TruSeq DNA Library Kit using a multiplex identifier adaptor index. Illumina sequencing was conducted on a HiSeq with 100‐bp paired‐end reads. Five million of the resulting reads were analyzed with the program PAL_FINDER_v0.02.03 (Castoe et al., 2012) to extract those reads that contained di‐, tri‐, tetra‐, penta‐, and hexanucleotide microsatellites. Once positive reads were identified, they were batched to a local installation of the program Primer3 (version 2.0.0) for primer design. To avoid issues with copy number of the primer sequence in the genome, loci for which the primer sequences only occurred one or two times in the five million reads were selected. Forty‐eight potential loci that met this criterion were chosen. One primer from each pair was modified on the 5′ end with an engineered sequence (CAG tag 5′‐CAGTCGGGCGTCATCA‐3′) to enable use of a third primer in the PCR (identical to the CAG tag) that was fluorescently labeled. The sequence GTTT was added to primers without the universal CAG tag addition.

The 48 potential primer pairs were tested for amplification and polymorphism using DNA obtained from eight individuals. PCR amplifications were performed in a 12.5 μl volume (10 mM Tris [pH 8.4], 50 mM KCl, 25.0 μg/ml BSA, 0.4 μM unlabeled primer, 0.04 μM tag labeled primer, 0.36 μM universal dye‐labeled primer, 3.0 mM MgCl_2_, 0.8 mM dNTPs, 0.5 units JumpStart Taq DNA Polymerase [Sigma], and ~20 ng DNA template) using an Applied Biosystems GeneAmp 9700. Touchdown thermal cycling programs (Don, Cox, Wainwright, Baker, & Mattick, 1991) encompassing a 10°C span of annealing temperatures ranging between 65 and 55°C (TD65) were used for all loci. Touchdown cycling parameters consisted of an initial denaturation step of 5 min at 95°C followed by 20 cycles of 95°C for 30 s, highest annealing temperature (decreased 0.5°C per cycle) for 30 s, and 72°C for 30 s; and 20 cycles of 95°C for 30 s, lowest annealing temperature for 30 s, and 72°C for 30 s, and a final extension at 72°C for 5 min. PCR products were run on an ABI 3130xl sequencer and sized with Naurox size standard prepared as described in DeWoody et al. (2004), except that unlabeled primers started with GTTT. Results were analyzed using GeneMapper version 3.7 (Applied Biosystems).

We further assessed the variability of ten polymorphic loci (Almi 8, Almi 19, Almi 26, Almi 30, Almi 32, Almi 34, Almi 39, Almi 40, Almi 46, and Almi 47) across all adult individuals using the same conditions described above with a touchdown protocol and highest annealing temperature of 58°C. Allele frequencies for these ten loci were estimated using all adults captured during the course of the study. We estimated the number of alleles per locus (*k*), observed and expected heterozygosity (*H*_o_ and *H*_e_), mean polymorphic information content (PIC), the nonexclusion probability for the first parent (NE‐1P), the nonexclusion probability for the second parent (NE‐2P), and the nonexclusion probability for the parent pair (NE‐PP) with Cervus 3.0.7 (Kalinowski et al., 2007). Tests for deviations from Hardy–Weinberg equilibrium (HWE) and for linkage disequilibrium were conducted using GENEPOP v4.0 (Rousset, 2008). Characteristics of the loci are provided in Table S1. After determining that these 10 loci would provide the power needed for parentage analyses, we genotyped 1,657 hatchlings across all 10 loci using the same conditions.
