# Supplementary material for: Mating dynamics and multiple paternity in a long‐lived vertebrate
Source: Ecol Evol. 2019 Sep 5;9(18):10109–21. doi: 10.1002/ece3.5438 (PMC6787947; doi:10.1002/ece3.5438)
Supplement: Supplementary file 1 [file ECE3-9-10109-s001.docx]

**SI Table 1.** The forward and reverse primer sequence used for each microsatellite loci used in the analysis as well as the repeated sequence motif for each loci. Each sequence includes the CAG forward primer and the pigtailed reverse primer.

| **Loci** | **Primer Sequence 5' -> 3'** | **Motif** |
| --- | --- | --- |
| Almi 8 | **F**:CAGTCGGGCGTCATCACCTTAATTATGAATTATCCGGAGGG **R:** GTTTAATCCTCCCTGACATTTCCC | ATCT (56) |
| Almi 19 | **F:**CAGTCGGGCGTCATCAGCAGACTCTAGAGCATTTAGAATAGTCC **R:** GTTTCAAGTCAGGTTCACTTGTATCTAAACTAGC | ATCT (52) |
| Almi 26 | **F:**CAGTCGGGCGTCATCAGAACCAGTAAGTGCCCTCCC **R:** GTTTCGAAACAGAAGTCACACATCCC | ATCT (68) |
| Almi 30 | **F:**CAGTCGGGCGTCATCATTAGACCCTGTTGCCCATCC **R:** GTTTGCCCTCTTCTTCATCATGCC | ATCT (60) |
| Almi 32 | **F:**CAGTCGGGCGTCATCATGTCTGGCCTGGAAAGATCC **R:** GTTTGGGAGTACCTGCCTGTTCCC | ATCT (56) |
| Almi 34 | **F:** CAGTCGGGCGTCATCAGGAGTGCAGATGTCCAGG **R:** GTTTGTTCGGACCAGCAGCACC | ATCT (48) |
| Almi 39 | **F:**CAGTCGGGCGTCATCAAGTCTCCCTACACACAGGG **R:** GTTTGCAGTCAGGGACAGACTACC | ATCT (52) |
| Almi 40 | **F:**CAGTCGGGCGTCATCAGGCTCTGTGCATCTTGCTCC **R:** GTTTGGTATGGGATGCTAAGCCC | ATCT (64) |
| Almi 46 | **F:**CAGTCGGGCGTCATCATTTGTTTCCTATCTTTCCTCCC **R:** GTTTGAGAACACTTCAACGTTTCC | ATCT (56) |
| Almi 47 | **F:**CAGTCGGGCGTCATCAGGAGTTCTCTGATGATCCTATCCC **R:** GTTTATTGGAGGATGTCATTGGG | ATCT (64) |

**SI Table 2.** The nests in which mate fidelity occurred.

| **Year** | **Nest ID** | **Female** | **Male** |
| --- | --- | --- | --- |
| 2016 | 13_2016 | CF-35 | CG-18 |
| 2017 | 35_2017 | CF-35 | CG-18 |
| 2013 | 7_2013 | BE-02 | BF-05 |
| 2015 | 3_2015 | BE-02 | BF-05 |
| 2012 | 3_2012 | CF-40 | CI-28 |
| 2016 | 1_2016 | CF-40 | CI-28 |
| 2017 | 24_2017 | CF-40 | CI-28 |
